# Tandem integration of circular plasmid contributes significantly to the expanded mitochondrial genomes of the green-tide forming alga *Ulva meridionalis* (Ulvophyceae, Chlorophyta)

**DOI:** 10.3389/fpls.2022.937398

**Published:** 2022-08-05

**Authors:** Feng Liu, Hongshu Wang, Wenli Song

**Affiliations:** ^1^CAS Key Laboratory of Marine Ecology and Environmental Sciences, Institute of Oceanology, Chinese Academy of Sciences, Qingdao, China; ^2^Marine Ecology and Environmental Science Laboratory, Pilot National Laboratory for Marine Science and Technology (Qingdao), Qingdao, China; ^3^Center for Ocean Mega-Science, Chinese Academy of Sciences, Qingdao, China; ^4^Rongcheng Ecological Civilization Construction Coordination Center, Weihai, China

**Keywords:** mitochondrial genome, circular plasmid, DNA-directed RNA polymerase, *Ulva meridionalis*, Ulvophyceae, green tide

## Abstract

Comparative mitogenomics of *Ulva* species have revealed remarkable variations in genome size due to the integration of exogenous DNA fragments, the proliferation of group I/II introns, and the change of repeat sequences. The genus *Ulva* is a species-rich taxonomic group, containing a variety of green-tide forming algae. In this study, five complete mitogenomes of the green-tide forming macroalga, *Ulva meridionalis* R. Horimoto and S. Shimada, were assembled and compared with the available ulvophyceae mtDNAs. The main circular mitogenomes of *U. meridionalis* ranged from 82.94 to 111.49 kb in size, and its 111.49-kb mitogenome was the largest *Ulva* mitogenome sequenced so far. The expansion of *U. meridionalis* mitogenomes is mainly due to the tandem integration of a 5.36-kb mitochondrial circular plasmid (pUme), as well as the proliferation of introns. An intact DNA-directed RNA polymerase gene (*rpo*) was present in pUme of *U. meridionalis* and was then detected in two putative plasmids (pUmu1 and pUmu2) found in *Ulva mutabilis*. The observed integration of the circular plasmid into *U. meridionalis* mitogenomes seems to occur *via* homologous recombination, and is a more recent evolutionary event. Many highly homologous sequences of these three putative plasmids can be detected in the other *Ulva* mtDNAs sequenced thus far, indicating the integration of different mitochondrial plasmid DNA into the mitogenomes is a common phenomenon in the evolution of *Ulva* mitogenomes. The random incidence of destruction of plasmid-derived *rpo*s and open reading frames (*orf*s) suggests that their existence is not the original characteristic of *Ulva* mitogenomes and there is no selective pressure to maintain their integrity. The frequent integration and rapid divergence of plasmid-derived sequences is one of the most important evolutionary forces to shape the diversity of *Ulva* mitogenomes.

## Introduction

The species of genus *Ulva* Linnaeus 1753 (Ulvophyceae, Chlorophyta) have attracted much attention not only because of their potential economic value in food and pharmaceutical industry, but also because of their important ecological functions and effects. These green seaweeds widely distributed on the coasts of the world are important indicators to reflect the state of ecological and environmental health. Globally, these opportunistic *Ulva* species often accumulate large amounts of biomass due to eutrophication, resulting in large-scale green tides (Ye et al., 2011; Liu et al., 2013; Wang et al., 2015, 2019). In the past 5 years from 2017 to 2021, *Ulva meridionalis* R. Horimoto and S. Shimada has proliferated and grown rapidly every summer in the Sakura Lake, Rongcheng, Shandong Province, China. The Sakura Lake is a semi-enclosed brackish inner lake, located at the tip of Shandong Peninsula, China, and extends to the Sanggou Bay which is connected to the Yellow Sea. This lake receives the freshwater runoffs and effluents from the local largest river, Gu River, and other small rivers, which carry the essential dissolved plant nutrients from agricultural, industrial, and municipal activities and cause a worsening trend of eutrophication. The large *U. meridionalis* biomass had a strong negative impact on the local landscape and ecosystem, and thousands of tons of biomass could only be salvaged manually. This brackish alga with tubular, winkled or lubricous thalli was taxonomically named in 2011, and mainly inhabits estuaries and marshes by the sea in Japan and China (Horimoto et al., 2011; Xie et al., 2020; Liu J. et al., 2022). This alga has attracted much attention because of its rapid growth ability (e.g., Hiraoka et al., 2020; Tsubaki et al., 2020).

The species-rich macroalgal genus *Ulva* currently contains at least 99 taxonomically accepted species worldwide (Guiry and Guiry, 2022), as well as some unconfirmed cryptic species (Steinhagen et al., 2019a,b). *Ulva* species have high morphological diversity and plasticity at the intraspecific level (Gao et al., 2016; Steinhagen et al., 2019c), so accurate and reliable species identification often requires the use of common DNA markers (e.g., ITS, *rbc*L, *tuf*A, etc.) (Blomster et al., 2002; Liu et al., 2013; Fort et al., 2020). Organelle genome as a molecular marker can make us more accurately understand the concept of *Ulva* species and more comprehensively understand their genetic diversity and evolutionary relationships, which could not be done by a single or several DNA markers. Recently, organelle genomes of *Ulva* species showed a variety of obvious dynamic changes involving genome size, integration of exogenous DNA fragments, gene content, acquisition or loss of intron, genome rearrangement, and abundance of repeat sequence, at the interspecific and intraspecific level (Liu and Melton, 2021; Liu F. et al., 2022). Based on the phylogenetic analysis of organelle genome data, *Ulva* species are obviously divided into two independent genetic lineages (I and II) (Liu and Melton, 2021; Liu F. et al., 2022). In addition, nuclear genomes of two *Ulva* species, *Ulva mutabilis* and *Ulva prolifera*, have been sequenced and deposited in the GenBank database up to now (De Clerck et al., 2018).

In the last 10 years, mitogenomic data on *Ulva* have accumulated rapidly. Thus far, a total of 32 mitochondrial genomes (mitogenomes or mtDNAs) from 19 *Ulva* species have been sequenced and deposited in the GenBank database (Liu F. et al., 2022). The complete *Ulva* mitogenomes are circular molecules with the size ranging from 55.81 to 88.42 kb, and display great changes in genome size (Liu et al., 2017, 2020; Liu F. et al., 2022). The *Ulva* mitogenomes contain the same set of 62 core genes which are usually coded on one strand, while specific genes and open reading frames (*orf*s) vary greatly in quantity and sequence. It is worth noting that DNA-directed RNA polymerase genes (*rpo*s) are very commonly present in the *Ulva* mitogenomes, which are likely to be the origin of mitochondrial plasmid DNA, and many *rpo*s have been split into small segments in varying degrees due to multiple mutations (Liu F. et al., 2022). The introns in *Ulva* mitogenomes show drastic dynamic changes in the number, distribution and diversity at interspecific and intraspecific level, and none of introns is shared by all sequenced *Ulva* mitogenomes, indicating that the homing or “jumping” of group I/II introns occurred frequently in *Ulva* (Liu et al., 2017; Liu F. et al., 2022). The *Ulva* mitochondrial introns observed are mainly distributed at 29 insertion sites in seven genes (*atp1*, *cox1*, *cox2*, *nad3*, *nad5*, *rnl*, and *rns*) and usually harbor an intronic *orf* which encoded an LAGLIDADG homing endonuclease (LHE) or a GIY-YIG homing endonuclease (GHE) or a reverse transcriptase/maturase (RTM). Six types of group I/II introns have been found in *Ulva* mitogenomes, and in particular, the mitochondrial LHEs in group II introns have close relationships with that in group IB introns (Liu and Melton, 2021).

In addition to the main mitochondrial genomes, mitochondria of some fungi and plants harbor some smaller DNA molecules regarded as plasmid-like elements or true plasmids, which could be autonomously replicated (Handa, 2008; Lang, 2014). The true plasmids can be divided into three different categories: (1) linear plasmids that encode a DNA and/or an RNA polymerase, (2) circular plasmids that encode a DNA polymerase, and (3) linear or circular retroplasmids that encode a reverse transcriptase (Hausner, 2011; Baidyaroy et al., 2012). Mitochondrial plasmids show great diversity in sequence and structure, and many plasmids are species-specific in distribution pattern. Although most plasmids appear to be cryptic in nature, they seem to be involved in the evolution of mitogenomes and are related to mitochondrial instability in fungi and cytoplasmic male sterility (CMS) in plants (Hausner, 2003; Gualberto et al., 2014). More evidences showed that mitochondrial plasmid DNA could be integrated into the mitogenomes in fungi and plants, which caused the increase of mitogenome size (e.g., Allen et al., 2007; Formighieri et al., 2008). However, there is little knowledge on the mitochondrial plasmid in green algae thus far.

To understand the evolution of *Ulva* mitogenomes and the formation mechanism of mitogenome diversity, in this study, five complete mitochondrial genomes of the green-tide forming macroalga *U. meridionalis* (*Ume*) have been sequenced and compared with the available ulvophyceae mtDNAs deposited in the GenBank database.

## Materials and methods

### Sample collection and species identification

The drifting algal thalli of *Ulva meridionalis* R.Horimoto and S.Shimada were collected on 4 August 2021 in the Sakura Lake (37°7′30″–56″’N, 122°27′3″–50″E), Rongcheng, Shandong Province, China, when a green tide occurred in the lake due to the proliferation of *U. meridionalis* (*Ume*). These *Ulva* thalli were transported to laboratory in coolers (5–8°C) after collection. Five algal individuals (LF008, LF010, LF011, LF012, and LF018) were randomly selected from 60 identified samples, and named as *Ume1* to *Ume5*, respectively (Supplementary Figure 1). Algal thallus for each individual was cultured in a 9-cm diameter Petri dish containing 25-mL L1 medium with 0.5‰ GeO_2_, 50 μg/mL dipterex (Fengcheng Animal Medicine Co., Ltd, China) and a suite of antibiotics (per mL: 50 μg streptomycin, 66.6 μg gentamycin, 20 μg ciprofloxacin, 2.2 μg chloramphenicol, and 100 μg ampicillin) (Shibl et al., 2020). The culture of *U. meridionalis* was maintained at 18°C, 100–120 μmol photons m^–2^ s^–1^ in the photoperiod of 12 h light: 12 h darkness in a GXZ-380C temperature-controlled incubator (Ningbo Jiangnan, China).

Fresh algal tissue from each individual thallus was used for DNA extraction using a Plant Genome DNA Kit (DP305, Tiangen Biotech, Beijing, China) according to the manufacturer’s instructions. Species identification was performed based on phylogenetic analyses of two common DNA marker datasets (the nuclear ITS region including the 5.8S rDNA gene, and the chloroplast *rbc*L gene) (Hayden and Waaland, 2002; Liu et al., 2020). Primers sequences and polymerase chain reaction (PCR) amplification were used according to our previous study (Liu et al., 2013). Sequence datasets of our samples and other data from the GenBank database were aligned using MEGA 7.0 (Kumar et al., 2016). The maximum likelihood (ML) tree was constructed with 1,000 bootstrap replicates based on the Kimura two-parameter model (Tamura and Nei, 1993). The identification results confirmed that these five samples were *U. meridionalis* (Supplementary Figures 2, 3).

### DNA sequencing and mitogenome assembly

The DNA quality and concentration were checked using a NanoPhotometer spectrophotometer (Implen, CA, United States), and a Qubit 2.0 Fluorometer (Life Technologies, CA, United States), respectively. Qualified DNA samples were fragmented into 350 bp by Covaris S220 ultrasonic crater for library construction. The qualified libraries were sequenced on an Illumina NovaSeq platform (Illumina, United States) using paired-end sequencing, yielding about 10 Gb sequencing raw data of paired-end reads with 150 bp in length for each *U. meridionalis* sample. Clean data were obtained by trimming sequencing adapters and removing short or low-quality reads from the raw data. The complete mitochondrial genomes of *U. meridionalis* were constructed by the GetOrganelle v1.7.1 (Jin et al., 2020). The mitogenome of *Ulva prolifera* (KT428794) was used as a reference genome for assembly. The mitogenome assembly was examined by aligning reads using the MEM algorithm of BWA v0.7.17 (Li and Durbin, 2010). The VarScan v2.3.9 (Koboldt et al., 2009) and IGV v2.8.12 (Robinson et al., 2011) were used to examine mutation sites and to verify the assembly results, respectively.

### Annotation of mitochondrial genomes

Protein-coding genes (PCGs) were annotated by Open Reading Frame Finder at the National Center for Biotechnology Information (NCBI) website,^1^ and by aligning the homologous PCGs from the *Ulva* mtDNAs deposited in the GenBank database with the newly sequenced *U. meridionalis* mitogenomes. Transfer RNA genes (tRNAs) were searched for by reconstructing their cloverleaf structures using the tRNAscan-SE 2.0 software with default parameters (Chan et al., 2021). Ribosomal RNA genes (rRNAs) were identified by the RNAweasel Tool^2^ and by aligning the homologous rRNAs. The free-standing and intronic open reading frames (*orf*s) were found by Open Reading Frame Finder at the NCBI website. Intron insertion-sites were determined manually by aligning the intron-containing homologous genes including *atp1*, *cob*, *cox1*, *cox2*, *nad3*, *nad5*, *rnl*, and *rns*. The corresponding genes in the *Ulva compressa* (KY626327) mitogenome were used as a reference (Liu et al., 2020). Intron name was defined as host gene plus insertion site. The class and core structure of all these introns were determined using the RNAweasel Tool and Mfold (Zuker, 2003). The physical maps of the circular mitogenomes were generated by using Organellar Genome DRAW (OGDRAW) (Greiner et al., 2019).

### Blast searches of plasmid(-derived) sequences and phylogenetic analysis of RNA polymerase genes

The DNA-directed RNA polymerase gene (*rpo*) in mitochondrial circular plasmid of *U. meridionalis*, which we call pUme, was searched against the database of the 98.5 Mbp haploid genome of *Ulva mutabilis* Föyn (De Clerck et al., 2018), which has been regarded as a taxonomic synonym of *Ulva compressa* Linnaeus (Steinhagen et al., 2019a), with tblastn.^3^ We detected two contigs, WT279 and WT234, which contained only the mitochondrial homologous *rpo*s and *orf*s with their multiple tandem arrangement. Two putative circular plasmids named pUmu1 and pUmu2 were predicted and annotated using Open Reading Frame Finder at the NCBI website and the tRNAscan-SE 2.0 software. To conduct a thorough search for mitochondrial plasmid-like sequences in *Ulva* mitogenomes, the putative circular plasmid sequences (pUme, pUmu1, and pUmu2) were searched against the NCBI nucleotide database with blastn.

To avoid phylogenetic artifacts caused by convergent base composition, the phylogenetic tree was constructed based on the amino acid (aa) sequences of full-length *rpo*s found in *Ulva* mtDNAs and putative plasmids. Multiple sequence alignments of Rpos were conducted using ClustalX 1.83 with the default settings (Thompson et al., 1997). The phylogenetic relationships were inferred by using the Maximum Likelihood (ML) method based on the JTT matrix-based model (Jones et al., 1992) using MEGA 7.0 (Kumar et al., 2016). Initial tree(s) for the heuristic search were obtained by applying Neighbor-Join and BioNJ algorithms to a matrix of pairwise distances estimated using a JTT model. There was a total of 1,022 positions in the final dataset of Rpos.

### Genomic and phylogenomic analyses

Base composition of *U. meridionalis* mitogenomes and plasmid DNA was determined using MEGA 7.0 (Kumar et al., 2016). Differences and identity values of gene sequences were calculated by use of the BioEdit v7.1.9 software (Hall, 1999). Tandem repeats were detected with Tandem Repeats Finder using the default settings (Benson, 1999). Inverted repeats were identified with Inverted Repeats Finder using the default settings.^4^ The aa sequences of 32 genes including 30 PCGs and two conserved *orf*s shared by the ulvalean mitogenomes sequenced thus far (Supplementary Table 1) were subjected to concatenated alignments using ClustalX 1.83 with the default settings (Thompson et al., 1997). The evolutionary history was inferred by using the ML method based on the JTT matrix-based model (Jones et al., 1992). Initial tree(s) for the heuristic search were obtained automatically by applying Neighbor-Join and BioNJ algorithms to a matrix of pairwise distances estimated using a JTT model. There was a total of 10,526 positions in the final aa dataset. Phylogenomic analysis was conducted with 1,000 bootstrap replicates using MEGA 7.0 (Kumar et al., 2016).

## Results and discussion

### Variations of mitogenome size, gene repertoire, and intron content

**FIGURE 1:**
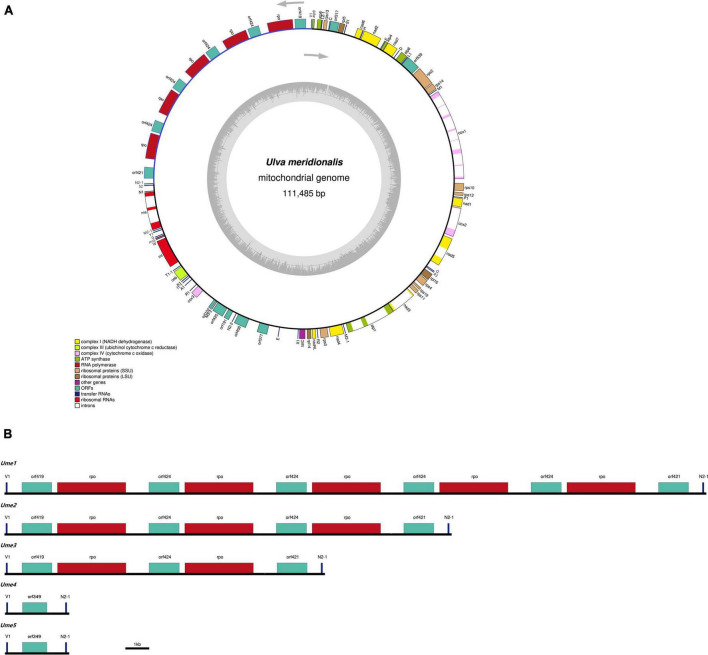
The mitochondrial genomes of *Ulva meridionalis*. **(A)** Gene map of the largest *U. meridionalis* mitochondrial genome (*Ume1*). Genes (*filled boxes*) shown on the outside of the map are transcribed counterclockwise. The smaller circle on the inside of the gene mapping shows a graph of the GC content. Genes are color coded for their functional gene group (legend bottom left). **(B)** Variations in the *trnV1-trnN2-1* intergenic region of five *U. meridionalis* mitogenomes (*Ume1*–*Ume5*) due to the tandem integration of circular plasmid (pUme).

**FIGURE 2:**
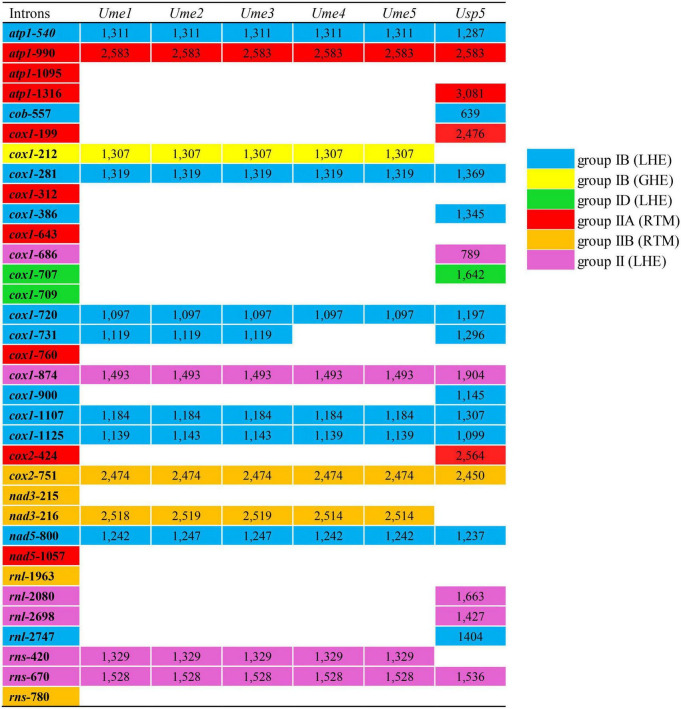
Insertion site, size and group of introns detected in five mitogenomes of *Ulva meridionalis* (*Ume1*–*Ume5*), as well as the mtDNA of *Ulva* sp. (MN853878) (*Usp5*) for comparative analysis. *Ulva prolifera* (MN853878) deposited in the GenBank database was corrected to *Ulva* sp. (MN853878), due to its wrong species name assignment. Intron name was defined as host gene plus insertion site which was determined by comparing homologous genes relative to the mitogenome of *U. compressa* (KY626327) (Liu F. et al., 2022). All insertion sites previously detected in the sequenced *Ulva* mitogenomes were listed in the first column. The intron *cox1*-214 is corrected to intron *cox1*-212.

Five mitochondrial genomes of *U. meridionalis* (*Ume*) had at least 5000 × depth of coverage (Supplementary Table 2 and Supplementary Figures 4–8) and mapped as complete circular molecules with the size ranging from 82.94 kb in *Ume4* and *Ume5* to 111.49 kb in *Ume1* (Table 1), displaying the most obvious genome expansion when compared with other *Ulva* mitogenomes sequenced (Melton et al., 2015; Suzuki et al., 2018; Liu et al., 2020; Liu F. et al., 2022). Their overall AT content of *U. meridionalis* mitogenomes is 66.15–66.22%, which is within the range (61.16–67.83%) of the *Ulva* mitogenomes reported (Liu F. et al., 2022). The 111.49-kb mitogenome of *Ume1* is the largest *Ulva* mitogenome sequenced so far (Figure 1A), and twice the smallest one which is the 55.81 kb mtDNA in *Ulva* sp. TM708 (Liu F. et al., 2022). The expansion of *U. meridionalis* mitogenomes is mainly due to the integration of plasmid-derived DNA fragments (Figure 1B), as well as the proliferation of introns (Figure 2).

**TABLE 1:** General features of mitochondrial genomes and circular plasmids in *Ulva meridionalis*.

General features	*Ulva meridionalis* (*Ume*)				
	
	*Ume1*	*Ume2*	*Ume3*	*Ume4*	*Ume5*
**Size (bp)**					
Mitogenome	111,485	100,796	95,439	82,944	82,944
Circular plasmid*	5,360	5,357	5,357	5,360	5,358
**A + T content (%)**					
Mitogenome	66.15	66.22	66.25	66.18	66.18
Core genes	65.65	65.65	65.65	65.65	65.65
Introns	61.96	61.97	61.97	61.55	61.55
Intergenic regions**	68.17	68.80	69.24	70.45	70.45
Circular plasmid	65.69	65.73	65.73	65.69	65.70
**Core genes** ***	64	64	64	64	64
PCGs/rRNAs/tRNAs/*orf*s/*rnpB*	30/3/28/2/1	30/3/28/2/1	30/3/28/2/1	30/3/28/2/1	30/3/28/2/1
**Introns**	14	14	14	13	13
Group I/group II	8/6	8/6	8/6	7/6	7/6
Intronic *orf*s	15	15	15	14	14
**Specific genes**	21	15	12	6	6
*rpo*s/*orf*s/tRNAs	5/11/2	3/9/2	2/8/2	0/6/2	0/6/2
**Copies of integrated plasmid**	5	3	2	0	0
**Genes in circular plasmid**	2	2	2	2	2
*rpo*s/*orf*s	1/1	1/1	1/1	1/1	1/1

*Circular plasmid included the integrated plasmid in Ume1 to Ume3, and the standalone plasmid in Ume4 and Ume5.

**The intergenic regions harbored specific genes including rpos, specific free-standing orfs and tRNA genes.

***Among these 64 core genes, 62 are common to all known Ulva mitogenomes. Two orfs in core genes represented conserved orfs, i.e. orf539 and orf317 in five mitogenomes of U. meridionalis.

The five *U. meridionalis* mitogenomes share the same set of 62 core genes as other *Ulva* mitogenomes (Liu F. et al., 2022), including 30 PCGs, three rRNAs (*rnl*, *rns*, and *rrn5*), 26 tRNAs, two conserved free-standing *orf*s, and one putative RNA subunit of RNase P (*rnpB*) (Table 1). One conserved *orf* (*orf219* in *U. meridionalis*) situated between *trnX1* and *rpl14* encodes the SecY-independent transporter protein TatC (gene name: *tatC* or *mttB*) (Turmel et al., 2016). A conserved EXXDSEL motif at the C-terminal fragment of TatC is shared by Ulvales and Ulotrichales, but not found in Oltmannsiellopsidales and Bryopsidales. The duplication mutation of *trnM3(cau)* was detected in the *U. meridionalis* mitogenomes (Table 2), which was not found in other *Ulva* mtDNAs, and resulted into two perfect copies of *trnM3(cau)* which located in the *nad4*-*atp1* and *cox3*-*trnE* intergenic regions, respectively. The function of *trnX1* is unknown, and the structure of *trnS3* is seriously degraded to loss of function in *U. meridionalis* mtDNAs. On top of that, two copies of specific *trnN2(guu)* found only in the *U. meridionalis* mitogenomes are a bit different from the core *trnN1(guu)* in the sequence of loops, especially DHU loop (Table 2), while the core *trnN1(guu)* is very conserved in all known ulvalean mitogenomes. These facts indicate that the *trnN2(guu)* is likely to appear in the *U. meridionalis* mitogenome through horizontal transfer rather than *trnN1(guu)* duplication.

**TABLE 2:** The aligned sequences of tRNA genes with duplication mutation in *U. meridionalis* mitogenomes.

tRNAs	Acceptor stem		DHU				Anticodon				TΨC			Acceptor stem
							
			Stem	Loop	Stem		Stem	Loop	Stem		Stem	Loop	Stem	
**M3-1**	GAGCAGC	TA	GCTC	AGATGGTA	GAGC	G	AGCGT	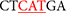	ACGCT	TGGTC	AGTAG	TTCGAAT	CTACT	GCTGCTT
**M3-2**	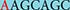	TA	GCTC	AGATGGTA	GAGC	G	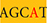	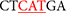	ACGCT	TGGTC	AGTAG	TTCGAAT	CTACT	GCTGCTT
**N1**	GCTTTTG	AA	GCTC	TGTGGTT	GAGC	G	CCAAG	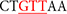	CTTGG	ATGAC	GCAGG	TTCGAAC	CCTGC	CAAAAGC
**N2-1**	GCTTTTG	AA	GCT	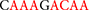	AGC	G	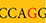	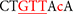	CTTGG	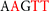	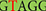	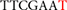	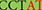	CAAAAGC
**N2-2**	GCTTTTG	AA	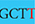	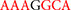	GAGC	G	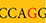	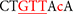	CTTGG	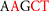	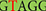	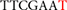	CCTGC	CAAAAGC

Shaded nucleotides indicated that bases could be paired.

The content of mitochondrial introns changed a bit at the intraspecific level in *U. meridionalis*, i.e., 14 introns in *Ume1* to *Ume3* and 13 in *Ume4* and *Ume5*. The intron *cox1*-731 was present in *Ume1* to *Ume3* but absent in *Ume4* and *Ume5* (Figure 2). These introns were located in six housekeeping genes, including *atp1* (two introns), *cox1* (seven or six), *cox2* (one), *nad3* (one), *nad5* (one), and *rns* (two). Most introns harbored an intact intronic *orf* encoded an LAGLIDADG homing endonuclease (LHE), or a GIY-YIG homing endonuclease (GHE), or a reverse transcriptase/maturase (RTM) (Dai et al., 2003; Haugen et al., 2005; Lambowitz and Zimmerly, 2011), but in intron *cox1*-212, the GHE gene was split into two parts, *orf152* and *orf158*. These introns can be divided into five types according to their secondary structure and intronic-encoding proteins, including group IB (LHE), group IB (GHE), group IIA (RTM), group IIB (RTM), and group II (LHE) (Figure 2). Five intron families, including intron *atp1*-1316, *cob*-557, *cox1*-686, *cox1*-707, and *cox1*-720, were discovered in *Ulva* for the first time, and other introns have been reported before (Liu F. et al., 2022).

The total intron length in *U. meridionalis* mitogenomes reached 20.52–21.65 kb, greatly exceeding the values of all reported *Ulva* mitogenomes (4.90–19.73 kb). The intronic AT content (61.55–61.97%) was significantly lower than that in mitogenomes (Table 1), indicating that introns as heterogeneous DNA showed distinct characteristics in base composition. Recently, a new record from the GenBank database on the 107.51-kb mitogenome of *Ulva* sp. (MN853878) showed that it contained a total of 22 introns (Figure 2), with a total intron length of 35.44 kb. The proliferation of introns greatly leads to the expansion of *Ulva* mitochondrial genomes, as was similar to that observed in mtDNAs of Ulotrichales and Bryopsidales (Pombert et al., 2004; Turmel et al., 2016; Zheng et al., 2018; Repetti et al., 2020).

### Mitochondrial circular plasmids in *Ulva*

**FIGURE 3:**
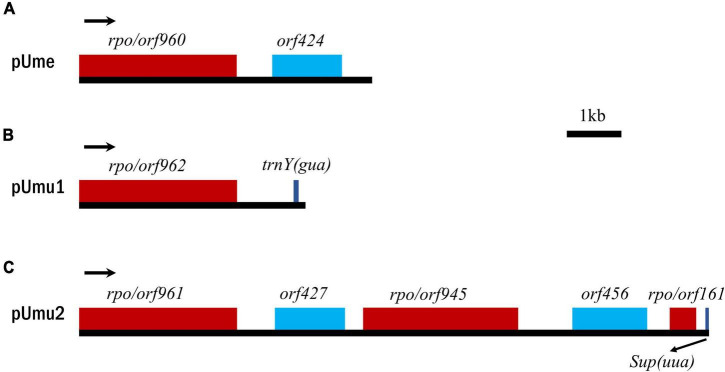
Comparison of gene maps of circular plasmids. **(A)** Circular plasmid (pUme) in *U. meridionalis*. **(B,C)** Two putative plasmids (pUmu1 and pUmu2) in *U. mutabilis*. Arrows indicate the direction of gene transcription.

Based on comparative analysis of these five *U. meridionalis* mitogenomes, we observed that five copies of a 5,360-bp plasmid-derived sequence were tandemly integrated into the *trnV1-trnN2-1* intergenic region in the mitogenome of *Ume1* (Figure 1), and three and two copies of its homolog with the size of 5,357 bp were integrated into the same target site in mtDNAs of *Ume2* and *Ume3*, respectively. However, none of such plasmid-derived sequence was present in mtDNAs of *Ume4* and *Ume5*. The copy number of integration plasmid was well supported by the similar depth of coverage between plasmid-derived sequence and the rest of mitochondrial genome (Supplementary Tables 2, 3 and Supplementary Figures 4–6). It is worth noting that this plasmid sequence was not found in mtDNAs of *Ume4* and *Ume5* (Supplementary Figures 7, 8), but a standalone mitochondrial circular plasmid was discovered in *Ume4* and *Ume5* (Figure 3A), with the size of 5,360 and 5,358 bp, respectively. This standalone plasmid in *Ume4* and *Ume5* is highly consistent with the integrated plasmid found in *Ume1* to *Ume3* in sequence, but slightly different in size mainly due to changes in the two polyC regions. The plasmid contigs have only 350 × and 1,000 × depth of coverage in *Ume4* and *Ume5*, respectively, which are significantly lower than that of mitochondrial contigs (9,100 × in *Ume4* and 13,500 × in *Ume5*) (Supplementary Table 4). These facts indicate that the circular plasmid exists in a free state in *Ume4* and *Ume5*, and the integrated plasmid in *Ume1* to *Ume3* should be from the free circular plasmid. Undoubtedly, it is reasonable that the circular plasmid exists in mitochondria of *Ume 1* to *Ume3* in both free and integrated states.

The free circular plasmid found in *Ume4* and *Ume5*, as well as the integrated plasmid in *Ume1* to *Ume3*, which we call pUme (Figure 3A), have the AT content of 65.69–65.73%, which is very similar to that of *U. meridionalis* mitogenomes (66.15–66.22%). The similarity of the AT content between pUme and *U. meridionalis* mitogenomes might be closely related to their coevolution and plasmid origin, as was markedly different from that observed in plants. Plant mitochondrial plasmids usually exhibit higher AT content than that of mitogenomes, and are suspected to have been acquired from a fungal donor (Handa, 2008; Beaudet et al., 2013; Warren et al., 2016).

The circular plasmids harbored one intact DNA-directed RNA polymerase (*rpo*) *orf960* and a specific *orf424* with unknown function based on blastp search. A small tandem repeat with the period size of 35 bp was located between *rpo* and *orf424*. The plasmid-encoded Rpo is in the single-chain RNA polymerase family of phage type that executes a specific-promoter transcription process similar to other multichain RNA polymerases (Cermakian et al., 1997; Yin et al., 2010; Warren et al., 2016). Previously, DNA and/or RNA polymerase genes were usually found in linear mitochondrial plasmids in fungi and plants, while some circular mitochondrial plasmids often contained DNA polymerase or reverse transcriptase genes (Hausner, 2003; Gualberto et al., 2014). It is worth noting that our study confirms that the *rpo* found in *U. meridionalis* mitogenomes is derived from the mitochondrial circular plasmid, and the *rpo*-containing circular plasmid represents a new class of mitochondrial plasmids, which expands our understanding of eukaryotic plasmid diversity.

Based on tblastn search of *rpo* gene in the 98.5 Mbp haploid genome of *U. mutabilis* (De Clerck et al., 2018), we detected two contigs, WT279 and WT234, which contained only the mitochondrial homologous *rpo*s and *orf*s with their multiple tandem arrangement. Their repeat units are *rpo*/*orf962-*ψ*trnY(gua)* and *rpo/orf961-orf427-rpo/orf945-orf456-rpo/orf161-*ψ*Supres(uua)* in WT279 and WT234, respectively (Figures 3B,C). A total of three full-length *rpo*s, and one incomplete *rpo*/*orf161* which is very similar to the homologous region in *rpo/orf945*, were detected in *U. mutabilis*. Considering that the complete sequences of these putative plasmids were not found in the mitogenome (WT177) of *U. mutabilis*, we infer that the reads assembled into these two contigs should belong to mitochondrial plasmid DNA. Two putative circular plasmids named pUmu1 and pUmu2 were constructed, with the size of 4,145 and 11,556 bp, respectively.

### Integration of circular plasmid into *Ulva* mitogenomes

**FIGURE 4:**
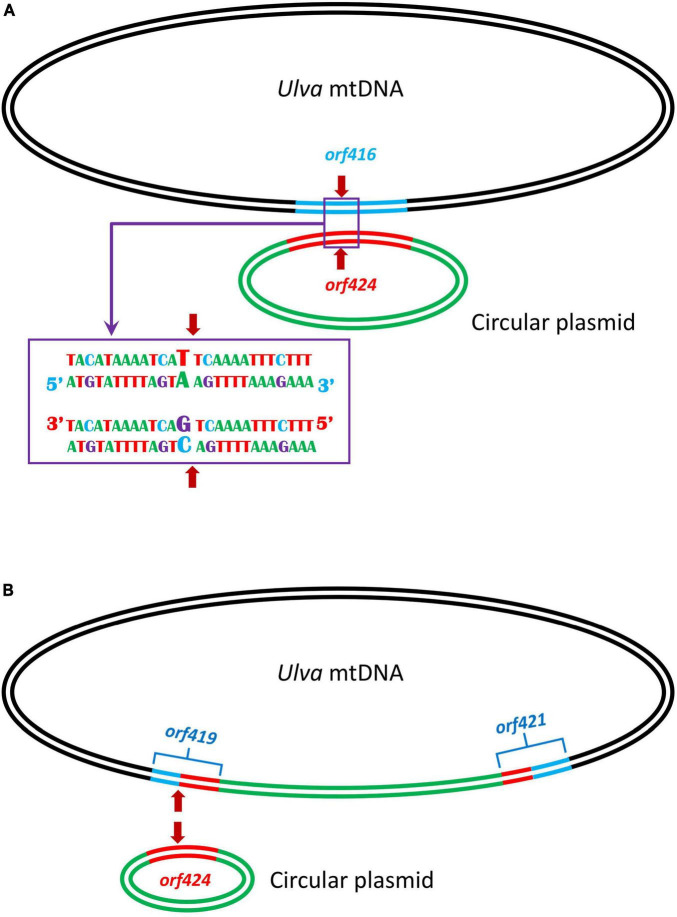
Model of tandem integration of circular plasmid into the *Ulva meridionalis* mitochondrial genome. **(A)** Integration of circular plasmid involving homologous recombination. A 152-bp sequence is shared by *orf416* in *Ulva* mtDNA and *orf424* in circular plasmid, and the homologous sequences are nearly completely identical, except that there is only one base difference at the integration site (the 43rd base of homologous sequences). The red arrow shows the integration site. **(B)** After the first integration of the circular plasmid, two chimeric *orf*s (*orf419* and *orf421*) were formed, and the subsequent integration of circular plasmid is more likely to occur in *orf419* which retained the only differential base at the integration site.

Comparisons of five *U. meridionalis* mitogenomes allowed us to model the integration process of circular plasmid into mtDNAs. The integration of circular plasmid should be *via* homologous recombination. A 152-bp homologous sequence is shared by *orf416* (or *orf349*) in *U. meridionalis* mtDNAs and *orf424* in standalone circular plasmid DNA, and these sequences are nearly completely identical, except that there is only one base difference at the integration site (the 43rd base of homologous sequences) (Figure 4A). The gtA(V) in *orf416* (or *orf349*) is instead of gtC(V) in *orf424*. After the first integration of the circular plasmid, two chimeric *orf*s (*orf419* and *orf421*) were formed. The subsequent integration of circular plasmid should occur at the same recombination site in *orf419* which retained the only differential base (Figure 4B), and generate two chimeric *orf*s which are identical to *orf419* and *orf424*, respectively. The observed integration of circular plasmid in *U. meridionalis* mitogenomes is a more recent evolutionary event, considering the sequence consistency and integrity between integrated and standalone plasmids.

**FIGURE 5:**
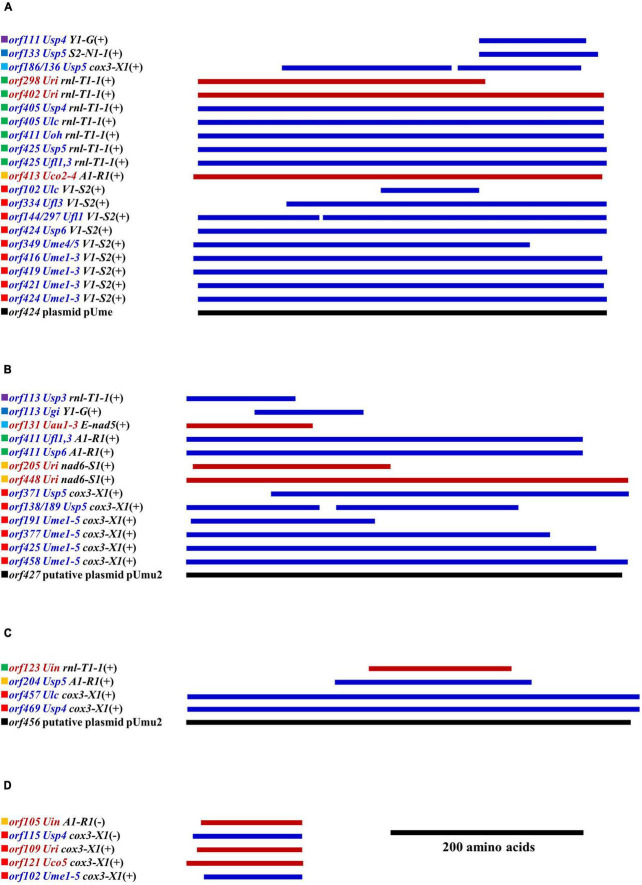
Schematic alignments of specific free-standing *orf*s detected in all sequenced mitogenomes (Supplementary Table 1) and putative circular plasmids of *Ulva* species. **(A)** Homologs of plasmid *orf424*. **(B)** Homologs of putative plasmid *orf427*. **(C)** Homologs of putative plasmid *orf456*. **(D)** Homologs of *orf102*. The solid squares with different colors represent different sources of specific *orf*s, including different intergenic regions (IRs) and putative plasmid. The *orfs* in blue font are from *Ulva* species in lineage I, and the *orf*s in red font are from *Ulva* species in lineage II. The *orfs* transcribed on the same strand as the core genes are marked with a positive sign (+), and the *orf*s transcribed on the minus strand are marked with a negative sign (–).

Many highly homologous sequences of these plasmids (pUme, pUmu1, and pUmu2) can be detected in mtDNAs of *Ulva* species but not found in mitogenomes of other ulvophyceae lineages (e.g., Ulotrichales, Bryopsidales, and Oltmannsiellopsidales) based on blastn search, but they showed varying levels of disruption and degradation in DNA sequence and different types of gene arrangement in structure, indicating the high diversity of plasmids and the differentiation of integration sites *via* homologous recombination. Sequence homology analysis of these *rpo*s and *orf*s (*orf424* in pUme, and *orf427* and *orf456* in pUmu2) showed that they were highly homologous with plasmid-derived *rpo*s and *orf*s previously found in *Ulva* mitogenomes (Figures 5A–C; Liu F. et al., 2022). However, we did not detect the ψ*trnY(gua)* in all sequenced *Ulva* mtDNAs, while the homologous suppressor tRNAs of ψ*Supres(uua)* were found in the *nad6-trnS1* intergenic region of *Ulva rigida* mtDNA and in the *trnR2-rps3* intergenic region of *Ulva flexuosa* mtDNAs. These facts showed that the integration of different mitochondrial plasmid DNA into the mitogenome is a common phenomenon in the evolution of *Ulva* mitogenomes.

Plasmid-derived sequence is one of the most important driving forces for the expansion of *Ulva* mitogenomes. In *U. meridionalis* mitogenomes, almost all specific free-standing *orf*s originate from plasmid. Although there is no evidence that the *orf102* which is located at the *cox3*-*trnX1* intergenic region of *U. meridionalis* mtDNAs is derived from plasmids, its homologs are present in different intergenic regions of other several *Ulva* mtDNAs (Figure 5D). The plasmid-derived DNA is even up to 33.96 kb in *Ume1*, accounting for 30% of the mitogenome, and appear at three intergenic regions, including *trnV1-trnN2-1*, *trnM3-2-trnN2-2*, and *trnN2-2-trnE*. Similar phenomena that the integration of circular or linear plasmids has greatly contributed to the increase of mitogenome size have also been reported in plants and fungi (e.g., Robison and Wolyn, 2005; Allen et al., 2007; Formighieri et al., 2008; Himmelstrand et al., 2014).

### DNA polymorphisms of *Ulva meridionalis* mitogenomes

Mitogenomes of *U. meridionalis* are readily distinguished by numerous DNA polymorphisms which have been characterized in this study (Supplementary Table 5), but their intraspecific polymorphisms are extremely low at the core genes. Nearly all core genes are identical in nucleotide sequence, and only two base substitutions were observed in two PCGs, including the 288 ggT → ggG transversion (amino acid: G, same sense mutation) which occurred only in *cox3* of *Ume5*, and the 567 ttA → ttT transversion (amino acid: L → F, missense mutation) which happened in *rps10* of *Ume1* to *Ume3* not in that of *Ume4* and *Ume5*. In addition, a 1-bp (A) deletion mutation occurred in the intronic *orf575* (RTM) of intron *nad3*-216 in *Ume1*, leading to premature termination of its homolog (*orf250*). A 2-bp (AT) deletion mutation which happened in the latter part of the free-standing *orf416* caused the early termination of its homolog (*orf349*) in *Ume4* and *Ume5*. Intraspecific polymorphisms of five *U. meridionalis* mitogenomes are significantly high at the intergenic regions and intronic non-coding regions. The divergence in length and sequence of intergenic regions in *U. meridionalis* mitogenomes was mainly caused by frequent insertion of foreign DNA fragments (e.g., plasmid DNA), rapid accumulation of multiple mutations, and dynamic fluctuation of repeat sequences (Supplementary Table 5), indicating the intergenic regions showed a rapid rate of DNA sequence evolution. There is a mutation hot spot region in the *trnE-trnX1* intergenic region, which can be divided into two DNA polymorphisms, i.e., type I in *Ume1* to *Ume3* and type II in *Ume4* and *Ume5* (Supplementary Table 5). The insertion of a specific DNA fragment occurred in the *trnA1-trnR1* intergenic region of the mitogenomes of *Ume1* to *Ume3*, but this DNA fragment is not present in mtDNAs of *Ume4* and *Ume5* as well as all other *Ulva* mtDNAs. The integrated DNA fragment with the size of 628 bp contains high A + T content (75.64%). A 7-bp sequence (CCTTTGC) is shared by the *U. meridionalis* mtDNAs and integrated DNA fragment, which should have function in the integration of this fragment.

All genes are coded on one strand in *U. meridionalis* mitogenomes, and the order of core genes is almost the same as that of other *Ulva* mitogenomes (Melton et al., 2015; Cai et al., 2018; Liu et al., 2020), except for the transposition of *trnE(uuc)* from the intergenic region of *trnQ-nad5* to that of *orf377-trnX1* (Figure 1A). We observed that some plasmid-derived *rpo*s and/or *orf*s sometimes encoded on another strand (Liu F. et al., 2022), which seems to depend on the integration direction of the plasmid DNA. This rarely occurs in core genes, but there is one exception. We previously found that large differences in the content of small inverted/tandem repeats can be observed in *U. compressa* mitogenomes at the intraspecific level, and the integration of small mobile inverted repeats has been shown to produce genomic rearrangements, which caused a gene cluster consisting of eight genes coding on another strand in one mitogenome of *U. compressa* (Liu et al., 2020). In addition to the repeats in the integrated plasmid sequence, five *U. meridionalis* mitogenomes shared almost the same set of small inverted/tandem repeats, most of which were present in intergenic regions. Only one tandem repeat with the period size of 16 bp appeared only in the *orf421-trnN1-2* intergenic region of *Ume2* and *Ume3*, due to a mutation event of this repeat sequence.

### Phylogenetic and phylogenomic analyses

**FIGURE 6:**
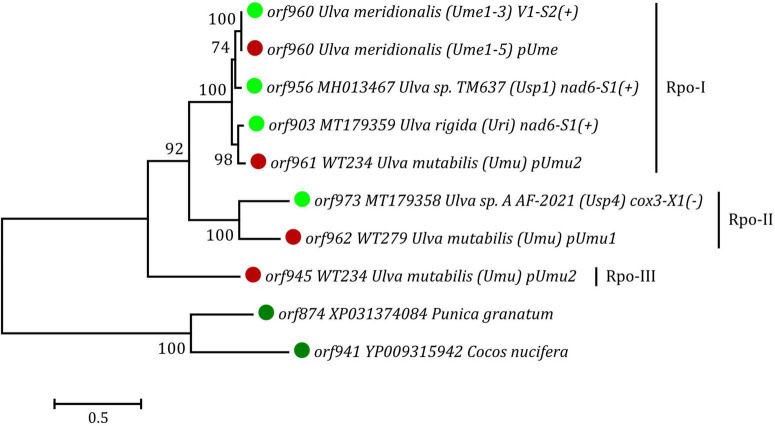
Phylogenetic tree based on Maximum Likelihood (ML) analysis of amino acid (aa) sequences of the full length Rpo proteins found in *Ulva* mitogenomes and putative plasmids. The ML analysis was performed with 1,000 bootstrap replicates using MEGA 7.0. The bootstrap support values greater than 70% were displayed at branches. Branch lengths were proportional to the amount of sequence change, which were indicated by the scale bar below the trees. The tree was rooted with two Rpo proteins from *Punica granatum* (XP031374084) and *Cocos nucifera* (YP009315942), respectively.

The ML phylogenetic analyses were conducted and restricted to full-length plasmid(-derived) Rpos, because many of these split Rpos found in *Ulva* mtDNAs are too short and divergent to provide comprehensive phylogenetic signals due to their rapid evolution. The Rpo dataset, including the newly discovered Rpos in circular plasmids and mitogenomes of *U. meridionalis*, and three Rpos found in two putative plasmids of *U. mutabilis*, as well as three full-length Rpos we previously reported in *Ulva* mtDNAs, represents a Chlorophyta-specific lineage, which is far away from other eukaryotic lineages (Liu F. et al., 2022). These full-length Rpos were clearly resolved into three different clades (Rpo-I, II and III), and each clade contained one of three copies of Rpo from two putative plasmids in *U. mutabilis* (Figure 6), indicating that the specific *Ulva* lineage of Rpo family showed great diversity, even at the intraspecific level. However, our understanding of these *rpo* genes in green algae is only the tip of the iceberg. Protein homologs of mitochondrial plasmid *rpo*s and *orf*s are very common in different intergenic regions of *Ulva* mitogenomes (Figure 5; Liu F. et al., 2022), indicating that the mitochondrial plasmid could be frequently integrated into many different target sites *via* horizontal transfer in the evolution of *Ulva* mitogenomes. These plasmid-derived sequences have rapidly diverged in *Ulva* mtDNAs, leaving numerous plasmid-derived genes or their remnants in the form of full-length or split *rpo*s and *orf*s, as well as large intergenic regions, due to multiple mutations that introduce internal stop codons, or insertions or deletions that result in frameshifts or loss of conserved domains. The random incidence of destruction of plasmid-derived *rpo*s and *orf*s suggests that their existence is not the original characteristic of *Ulva* mitogenomes, and there is no selective pressure to maintain their integrity in mitogenomes (Robison and Wolyn, 2005). These mobile *rpo*s and *orf*s are significantly different from the core genes in evolution, and could not be used to determine phylogenetic relationships of *Ulva* species.

**FIGURE 7:**
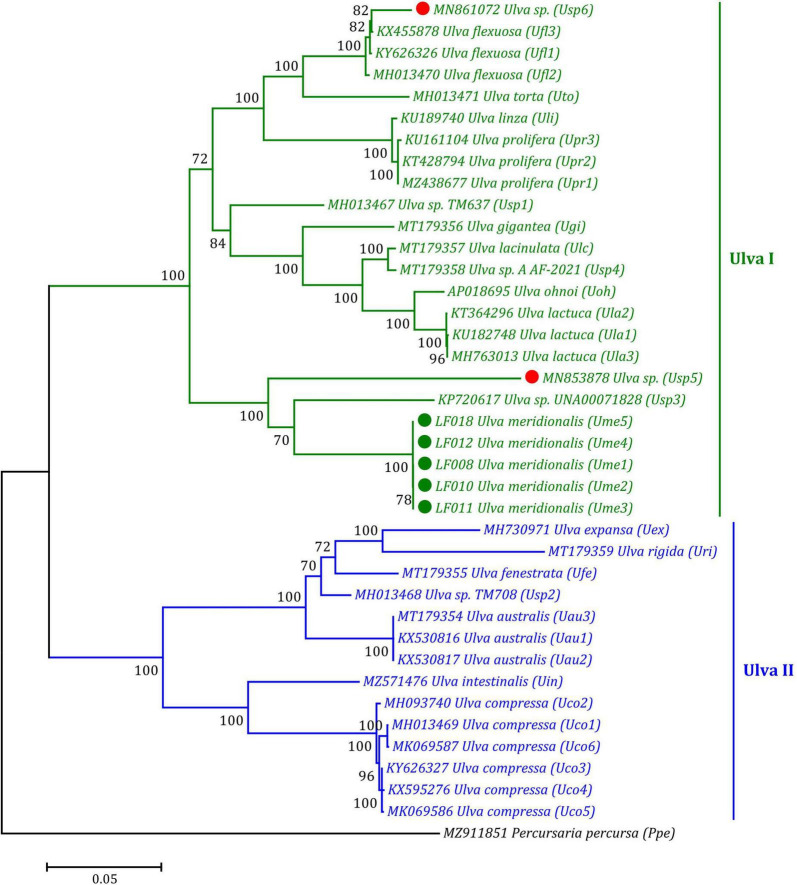
Phylogenomic tree based on Maximum Likelihood (ML) analysis of the amino acid (aa) sequences of 32 genes in 39 ulvalean mitogenomes. The ML analysis was performed with 1,000 bootstrap replicates using MEGA 7.0. The bootstrap support values greater than 70% were displayed at branches. Branch lengths were proportional to the amount of sequence change, which were indicated by the scale bar below the trees. *Ulva prolifera* (MN853878) and *Ulva meridionalis* (MN861072) deposited in the GenBank database were corrected to *Ulva* sp. (MN853878) and *Ulva* sp. (MN861072), respectively, due to their wrong species name assignment. The tree was rooted with *Percursaria percursa* (MZ911851) as an outgroup.

Based on the ML phylogenomic analysis of the aa sequences of 32 genes including 30 PCGs and two conserved *orf*s from the 39 ulvalean mtDNAs, *Ulva* species robustly clustered into two primary branches representing two *Ulva* genetic lineages, I and II, displaying the evolutionary nature of double crown radiation in the phylogeny and speciation of the *Ulva* group, as is consistent with our previous findings based on phylogenomics analysis of chloroplast genes (Liu and Melton, 2021; Liu F. et al., 2022). In the *Ulva* lineage I, *U. meridionalis* (*Ume1*–*Ume5*) first clustered with *Ulva* sp. (KP720617) and *Ulva* sp. (MN853878) to form an independent subclade with high support value (100%) (Figure 7). An incomplete mitogenome (MN861072) from one *Ulva* sample designated as *U. meridionalis* previously has been sequenced (Kang et al., 2020), which lacks the region from *rps10* to *cox1*. However, our result of phylogenomic analysis showed that this *Ulva* sample was closely related to *U. flexuosa* (Figure 7). The mitochondrial nucleotide (nt) sequences of some PCGs (*rps14*, *rpl5*, *atp9*, *tatC*, and *rps11*), two conserved *orf*s (*orf505* and *orf315*) and some intergenic regions in this sample are completely consistent with that in *U. flexuosa*, while other PCGs appear to be chimeras of *U. flexuosa* (KX455878) and *Ulva* sp. (MN853878). These results suggest that the mitogenome of this sample is an abnormal chimera, which may represent a cryptic species which we named *Ulva* sp. (MN861072) in this study.

## Conclusion

The mitochondrial genomes of *Ulva* species exhibit high diversity at the interspecific and intraspecific level due to their multiple variations which involves the integration of foreign DNA fragments, the acquisition or loss of introns, the dynamic change of repeat sequences, genome rearrangement, and multiple mutations (Liu et al., 2017, 2020; Liu F. et al., 2022). In this study, many new discoveries were unraveled in the evolution of *Ulva* mitogenomes. First, the expansion of *U. meridionalis* mitogenomes is mainly due to the tandem integration of mitochondrial circular plasmid as well as the proliferation of introns at the intraspecific level. The 111.49-kb *U. meridionalis* mitogenome is the largest *Ulva* mitogenome sequenced so far, even larger than some *Ulva* chloroplast genomes. Second, a 5.36-kb standalone mitochondrial circular plasmid (pUme) identified in *U. meridionalis* and two putative circular plasmids (pUmu1 and pUmu2) detected in *U. mutabilis* all harbor RNA polymerase genes (*rpo*s) which have only been found in mitochondrial linear plasmids in fungi and plants, which expand our understanding of eukaryotic plasmid diversity. Third, many highly homologous sequences of these plasmids (pUme, pUmu1, and pUmu2) can be detected in intergenic regions of *Ulva* mitogenomes sequenced so far, indicating that the integration of different mitochondrial plasmid DNA into the mitogenome is a common phenomenon in the evolution of *Ulva* mitogenomes. Fourth, these plasmid-derived *rpo*s and *orf*s have rapidly diverged and degenerated in *Ulva* mtDNAs, displaying markedly different evolution patterns from the core genes. The frequent integration and rapid divergence of plasmid-derived sequences greatly shape the diversity of *Ulva* mitogenomes at the intraspecific level. Finally, the ML phylogenomic analysis clearly depicted the evolutionary nature of double crown radiation in the phylogeny and speciation of the *Ulva* group.

## Data availability statement

The data presented in the study are deposited in the GenBank database, accession number ON402236 - ON402240. Data is publicly available by the following links: https://www.ncbi.nlm.nih.gov/nuccore/ON402236, https://www.ncbi.nlm.nih.gov/nuccore/ON402237, https://www.ncbi.nlm.nih.gov/nuccore/ON402238, https://www.ncbi.nlm.nih.gov/nuccore/ON402239, and https://www.ncbi.nlm.nih.gov/nuccore/ON402240.

## Author contributions

FL designed the study, performed the analysis, and wrote the manuscript. FL, HW, and WS performed the experiments. All authors have read and approved the final version of the manuscript.

## Footnotes

1
https://www.ncbi.nlm.nih.gov/orffinder

2
https://megasun.bch.umontreal.ca/cgi-bin/RNAweasel/RNAweaselInterface.pl

3
https://bioinformatics.psb.ugent.be/orcae/overview/Ulvmu

4
http://tandem.bu.edu/cgi-bin/irdb/irdb.exe
