# Supplementary figures and images for: Tandem integration of circular plasmid contributes significantly to the expanded mitochondrial genomes of the green-tide forming alga Ulva meridionalis (Ulvophyceae, Chlorophyta)
Source: Front Plant Sci. 2022 Aug 5;13:937398. doi: 10.3389/fpls.2022.937398 (PMC9389341; doi:10.3389/fpls.2022.937398)

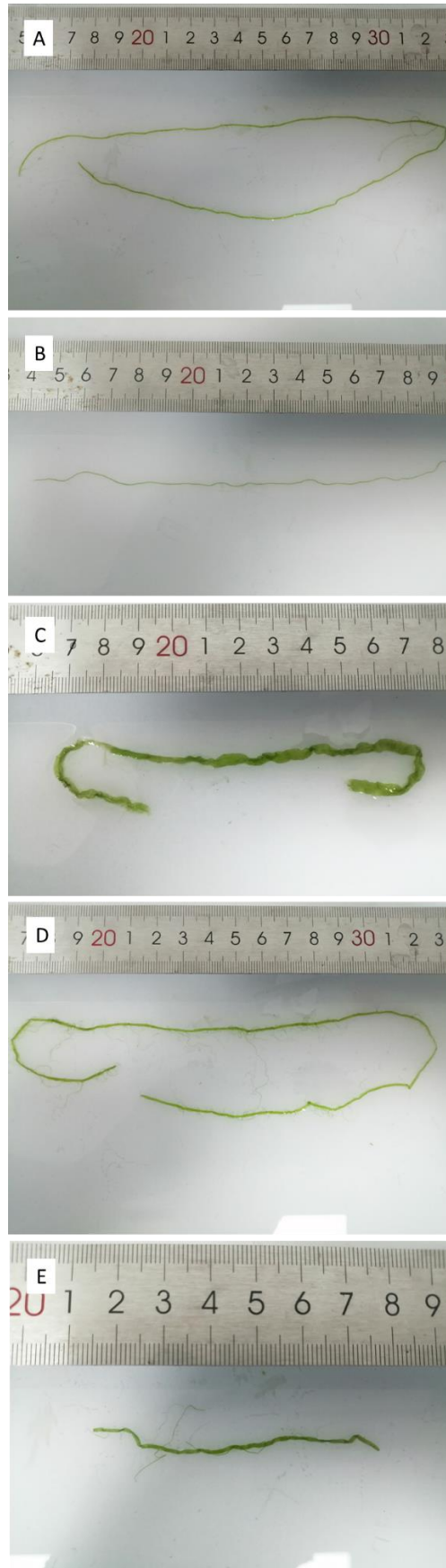

**Fig. S1** Morphology of five *U. meridionalis* samples. (A) LF008: *Ume1*. (B) LF010: *Ume2*. (C) LF011: *Ume3*. (D) LF012: *Ume4*. (E) LF018: *Ume5*.

Supplement: Supplementary file 1 [file Data_Sheet_1.PDF]
