# Supplementary material for: Tandem integration of circular plasmid contributes significantly to the expanded mitochondrial genomes of the green-tide forming alga Ulva meridionalis (Ulvophyceae, Chlorophyta)
Source: Front Plant Sci. 2022 Aug 5;13:937398. doi: 10.3389/fpls.2022.937398 (PMC9389341; doi:10.3389/fpls.2022.937398)
Supplement: Supplementary file 3 [file Data_Sheet_3.PDF]

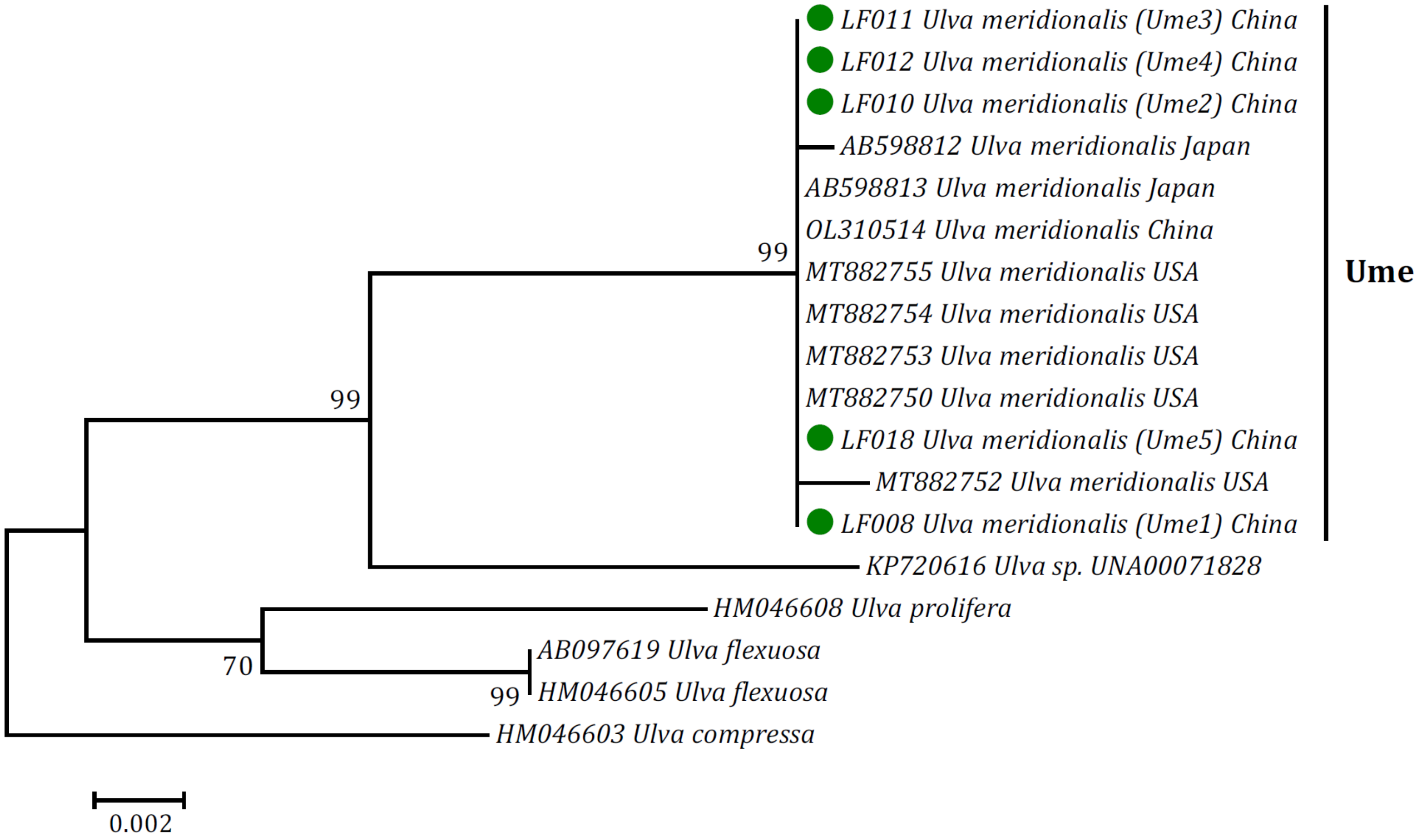

Fig. S3 Phylogenetic tree constructed from analyses of the chloroplast *rbc*L gene. The numbers at internal nodes are bootstrap values greater than 70% for 1000 replicates in the maximum-likelihood (ML) analysis. Branch lengths are proportional to the amount of sequence change, which are indicated by the scale bar below the tree.
