# Supplementary material for: Tandem integration of circular plasmid contributes significantly to the expanded mitochondrial genomes of the green-tide forming alga Ulva meridionalis (Ulvophyceae, Chlorophyta)
Source: Front Plant Sci. 2022 Aug 5;13:937398. doi: 10.3389/fpls.2022.937398 (PMC9389341; doi:10.3389/fpls.2022.937398)
Supplement: Supplementary file 6 [file Data_Sheet_6.PDF]

(A) 0 - 35 kb, start from *cox1*

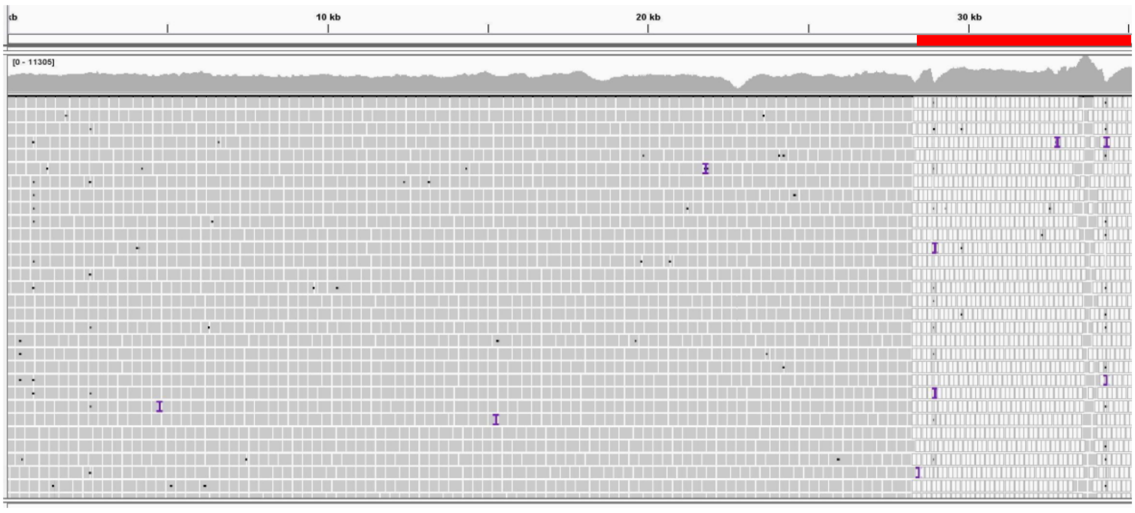


(B) 30 - 65 kb

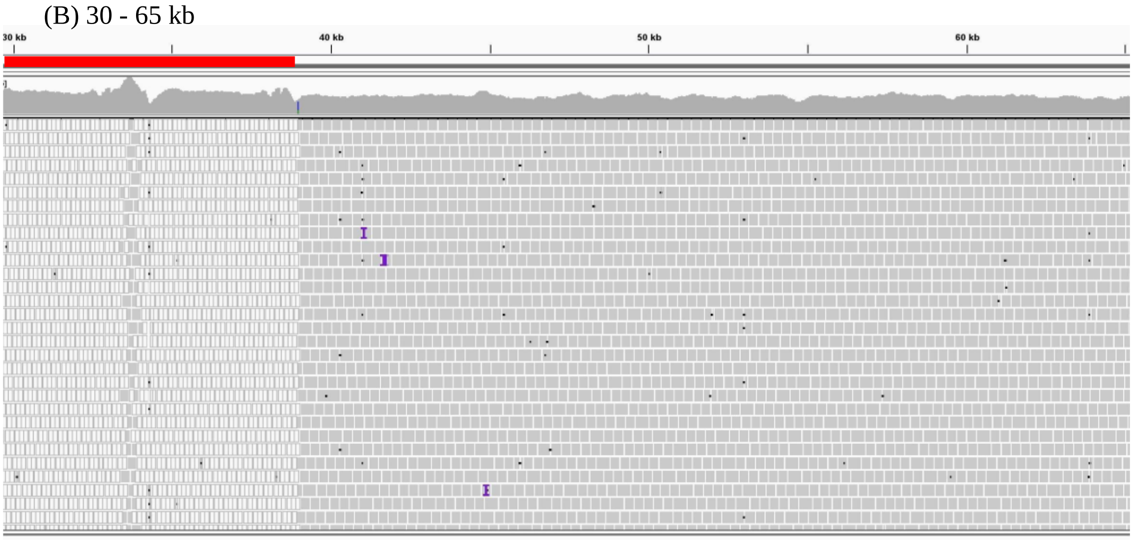


(C) 60 - 95.44 kb

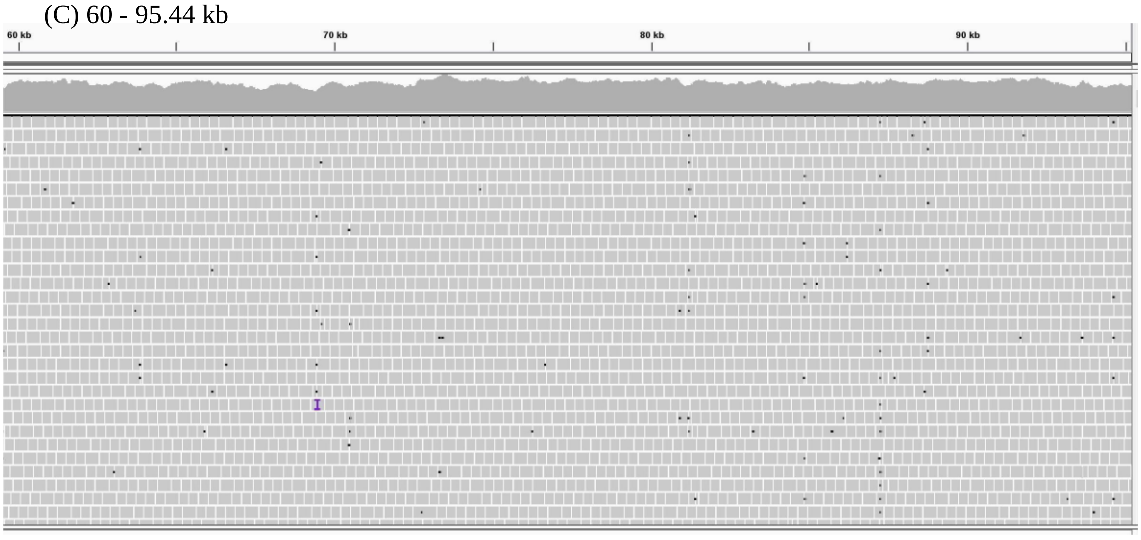


Fig. S6 Mapping result on the *Ume3* mitogenome based on IGV v2.8.12 software to show the relationship between read depth and mtDNA position. The red line represents the region of plasmid tandem integration.
