# Supplementary material for: Tandem integration of circular plasmid contributes significantly to the expanded mitochondrial genomes of the green-tide forming alga Ulva meridionalis (Ulvophyceae, Chlorophyta)
Source: Front Plant Sci. 2022 Aug 5;13:937398. doi: 10.3389/fpls.2022.937398 (PMC9389341; doi:10.3389/fpls.2022.937398)
Supplement: Supplementary file 9 [file Data_Sheet_9.PDF]

Table S1 The sequenced ulvalean mitogenomes used for comparative analysis and phylogenetic analysis in this study.

Lineage	Species	Abbr.	Accession number	Size (bp)
	Ulva sp. (*Ulva prolifera*) *	*Usp5*	MN853878	107,512
	Ulva torta	*Uto*	MH013471	65,772
	Ulva flexuosa	*Ufl1*	KY626326	71,527
	Ulva flexuosa	*Ufl2*	MH013470	63,526
	Ulva flexuosa	*Ufl3*	KX455878	71,545
	Ulva sp. (*Ulva meridionalis*) *	*Usp6*	MN861072	>62,887
	Ulva prolifera	*Upr1*	MZ438677	63,843
	Ulva prolifera	*Upr2*	KT428794	63,845
	Ulva prolifera	*Upr3*	KU161104	61,962
Ulva I	*Ulva linza*	*Uli*	KU189740	70,858
	Ulva lactuca	*Ula1*	KU182748	62,021
	Ulva lactuca	*Ula2*	KT364296	61,614
	Ulva lactuca	*Ula3*	MH763013	>61,125
	Ulva ohnoi	*Uoh*	AP018695	65,326
	Ulva lacinulata	*Ulc*	MT179357	79,723
	Ulva sp. A AF-2021	*Usp4*	MT179358	88,318
	Ulva gigantea	*Ugi*	MT179356	66,743
	Ulva sp. TM637	*Usp1*	MH013467	67,506
	Ulva sp. UNA00071828	*Usp3*	KP720617	73,493
	Ulva intestinalis	*Uin*	MZ571476	68,139
	Ulva compressa	*Uco1*	MH013469	61,700
	Ulva compressa	*Uco2*	MH093740	62,791
	Ulva compressa	*Uco3*	KY626327	62,477
	Ulva compressa	*Uco4*	KX595276	62,311
	Ulva compressa	*Uco5*	MK069586	>66,587
	Ulva compressa	*Uco6*	MK069587	67,021
Ulva II	*Ulva australis*	*Uau1*	KX530816	69,333
	Ulva australis	*Uau2*	KX530817	64,602
	Ulva australis	*Uau3*	MT179354	64,466
	Ulva sp. TM708	*Usp2*	MH013468	55,814
	Ulva fenestrata	*Ufe*	MT179355	59,026
	Ulva expansa	*Uex*	MH730971	64,143
	Ulva rigida	*Uri*	MT179359	88,416
Outgroup	*Percursaria percursa*	*Ppe*	MZ911851	>59,664

* *Ulva prolifera* (MN853878) and *Ulva meridionalis* (MN861072) deposited in the GenBank database were corrected to *Ulva* sp. (MN853878) and *Ulva* sp. (MN861072), respectively, due to their wrong species name assignment.
