# Supplementary material for: Tandem integration of circular plasmid contributes significantly to the expanded mitochondrial genomes of the green-tide forming alga Ulva meridionalis (Ulvophyceae, Chlorophyta)
Source: Front Plant Sci. 2022 Aug 5;13:937398. doi: 10.3389/fpls.2022.937398 (PMC9389341; doi:10.3389/fpls.2022.937398)
Supplement: Supplementary file 10 [file Data_Sheet_10.PDF]

Table S2 Assembly data of five *U. meridionalis* mitochondrial genomes.

Sources	Genome size (bp)	Total reads	Reads for mitogenome	Depth of coverage
Ume1	111,485	69,805,156	6,724,048	9047.0
Ume2	100,796	70,733,178	7,059,300	10505.3
Ume3	95,439	75,534,940	3,419,062	5373.7
Ume4	82,944	73,443,832	5,031,792	9099.7
Ume5	82,944	85,203,754	7,486,382	13538.7

Table S3 Assembly data of the integrated circular plasmid region in mtDNAs of *Ume1* - *Ume3*.

Sources	Size of integrated circular plasmid region (bp)	Reads	Depth of coverage
Ume1	26,800	1,556,082	8709.4
Ume2	16,071	1,205,506	11251.7
Ume3	10,714	473,566	6630.1

Table S4 Assembly data of the standalone mitochondrial circular plasmid pUme in *Ume4* and *Ume5*.

Sources	Size of standalone circular plasmid pUme (bp)	Reads	Depth of coverage
Ume4	5,360	12,662	354.3
Ume5	5,358	38,956	1090.6
