# Supplementary material for: Tandem integration of circular plasmid contributes significantly to the expanded mitochondrial genomes of the green-tide forming alga Ulva meridionalis (Ulvophyceae, Chlorophyta)
Source: Front Plant Sci. 2022 Aug 5;13:937398. doi: 10.3389/fpls.2022.937398 (PMC9389341; doi:10.3389/fpls.2022.937398)
Supplement: Supplementary file 11 [file Data_Sheet_11.PDF]

Table S5 The variations and DNA polymorphisms among the five *U. meridionalis* mitogenomes.

Positions	Mitogenomes					Depiction
	Ume1	***Ume2***	***Ume3***	***Ume4***	***Ume5***	
cox3	288T(96G)	288T(96G)	288T(96G)	288T(96G)	288G(96G)	Single base mutation. The 288 ggT → ggG transversion
rps10	567T(189F)	567T(189F)	567T(189F)	567A(189L)	567A(189L)	Single base mutation. The 567 ttA → ttT transversion
Intron *atp1*-990	C	C	C	T	T	Single base mutation
Intron *cox1*-731	+	+	+	-	-	Gain (+) or loss (-) of intron *cox1*-731
Intron *cox1*-1125	-	+	+	-	-	A 4-bp insertion (+)/deletion (-) mutation occurred upstream of the IEP gene
Intron *cox2*-751	C	G	G	C	C	Single base mutation
Intron *nad3*-216	+	+	+	-	-	A 5-bp repeat mutation occurred upstream of the IEP gene
	G	A	A	A	A	Single base mutation
	+	-	-	-	-	A 1-bp (A) deletion mutation occurred in the intronic *orf575* (RTM) of intron *nad3*-216 in *Ume1*, leading to premature termination of its homologue (*orf250*)
Intron *nad5*-800	-	+	+	-	-	Repeat mutation of five bases (TTTTT) in polyT region occurred downstream of the IEP gene
orf539-trnL1 intergenic	+	+	+	-	-	Repeat mutation of single base (A)

region (IR)						in polyA region
trnF-nad2 IR	A	T	T	A	A	Single base mutation
nad6-trnS1 IR	-	+	+	-	-	Repeat mutations of 4 bp (TATT) and 5 bp (TTTAA)
trnV1-trnN1-2 IR	A	C	C	A	A	Single base mutation
	+	-	-	-	-	A 11-bp insertion/deletion mutation
	-	+	+	+	+	A 16-bp insertion/deletion mutation
	G	C	C	G	G	Single base mutation
	5*5,360 bp	3*5,357 bp	2*5,357 bp	-	-	Integration of multiple circular plasmid DNA
	orf416	*orf416*	*orf416*	*orf349*	*orf349*	A 2-bp (AT) deletion mutation which happened in the latter part of the free-standing *orf416* caused the early termination of its homologue (*orf349*) in *Ume4* and *Ume5*.
	+	-	-	-	-	Repeat mutation of single base (T) in polyT region
	-	+	+	-	-	A 16 bp repeat mutation
trnS2-trnN1-1 IR	+	+	+	-	-	Repeat mutation of single base (A) in polyA region
trnW-rnl IR	-	+	+	+	+	Repeat mutation of single base (T) in polyT region
trnA1-trnR1 IR	+	+	+	-	-	Integration of a 628-bp DNA fragment
orf191-trnN1-3 IR	-	+	+	+	+	Repeat mutation of single base (T) in polyT region

orf377-trnE IR	T	A	A	T	T	Single base mutation
trnE-trnX1 IR	A	A	A	G	G	Single base mutation
	A	A	A	G	G	Single base mutation
	Type I	Type I	Type I	Type II	Type II	Mutation hot spot region
	T	T	T	C	C	Single base mutation
	C	C	C	A	A	Single base mutation
	C	C	C	A	A	Single base mutation
	G	G	G	A	A	Single base mutation
	T	T	T	C	C	Single base mutation
	T	T	T	C	C	Single base mutation
	G	G	G	T	T	Single base mutation
	A	A	A	G	G	Single base mutation
	T	T	T	G	G	Single base mutation
	-	-	-	+	+	Repeat mutation of single base (T) in polyT region
trnX1-tatC IR	T	A	A	A	A	Single base mutation
	A	T	T	T	T	Single base mutation
	A	T	T	T	T	Single base mutation
	T	A	A	A	A	Single base mutation
	A	T	T	T	T	Single base mutation
	A	T	T	T	T	Single base mutation
trnM3-1-atp1 IR	-	-	-	+	+	Repeat mutation of two bases (CC) in polyC region
	A	G	G	A	A	Single base mutation
trnQ-nad5 IR	A	A	A	G	G	Single base mutation

	+	+	+	-	-	A 4-bp repeat mutation (ATTT)
	A	A	A	T	T	Single base mutation
	+	-	-	-	-	Repeat mutation of single base (T) in polyT region
	T	T	T	C	C	Single base mutation
